# Visual Hebb Repetition Effects: The Role of Psychological Distinctiveness Revisited

**DOI:** 10.3389/fpsyg.2019.00017

**Published:** 2019-01-22

**Authors:** Andrew J. Johnson, Christopher Miles

**Affiliations:** Department of Psychology, Faculty of Science & Technology, Bournemouth University, Poole, United Kingdom

**Keywords:** Hebb repetition effects, visual memory, order memory, distinctiveness, serial position effect

## Abstract

Across two experiments we investigate the role of psychological distinctiveness on the Hebb repetition effect. In direct contradiction to Horton et al. (2008), Experiment 1 demonstrated the Hebb repetition effect for inverted faces. Importantly, the Hebb repetition effect was evident only when the filler and Hebb sequences comprised different items (no-stimulus-overlap) and was abolished when the filler and Hebb trials comprised the same items (full-stimulus-overlap). Experiment 2 further examined the impact of psychological distinctiveness on the Hebb repetition effect by comparing serial recall for upright unfamiliar-faces, inverted unfamiliar-faces, and abstract matrices. We demonstrate the visual Hebb repetition effect for stimuli that possess both purportedly high (upright faces) and low (inverted faces and matrices) levels of psychological distinctiveness. The findings of both experiments contradict the earlier claim (Horton et al., 2008) that stimuli possessing low levels of psychological distinctiveness do not show the visual Hebb repetition effect. However, we further highlight the importance of stimulus overlap between filler and Hebb sequences in determining the visual Hebb repetition effect. More generally, our findings emphasize that the Hebb repetition effect is a common feature of memory across different stimulus types.

## Introduction

The Hebb repetition effect refers to the gradual acquisition of sequence memory following surreptitious re-presentation of that sequence (Hebb, 1961). In a typical Hebb repetition procedure, participants undertake a series of immediate serial recall (ISR) trials for each of which participants must recall the sequence in its order of original presentation. Within a series, trials comprise both unique non-repeated (filler) sequences and a repeated Hebb sequence (typically re-presented every third trial). The Hebb repetition effect is evidenced by a gradual improvement in recall for the repeated sequence relative to the non-specific practice effects shown for the filler sequences.

The Hebb repetition effect has traditionally been studied within the context of verbal memory (e.g., Hebb, 1961; Cohen and Johansson, 1967a,b; Cunningham et al., 1984; Hitch et al., 2009; Oberauer and Meyer, 2009; Kalm and Norris, 2016), and the effect is linked with the process by which sequences of phonemes are transferred into lexical representations (e.g., see Cumming et al., 2003; Mosse and Jarrold, 2008; Page and Norris, 2009; Szmalec et al., 2009, 2011, 2012; Page et al., 2013; Smalle et al., 2016). Indeed, Page et al. (2013) showed that the verbal Hebb repetition effect exhibited three important features common to non-word learning, namely: (1) learning is observed when the interval between repetitions is increased, (2) participants are able to learn multiple Hebb sequences within an experiment, and (3) once learnt, memory for the Hebb sequence persists several months after the study.

Notwithstanding that evidence linking the Hebb repetition effect to phonological learning, a number of studies have shown that the effect is not dependent, exclusively, upon the use of verbal stimuli. For example, utilization of the phonological loop is not a pre-requisite for the Hebb repetition effect, because the effect maintains for recall of visual-verbal stimuli under conditions of concurrent articulation (Page et al., 2006; Hitch et al., 2009). Moreover, the Hebb repetition effect is evident for a range of non-verbal stimuli, e.g., unfamiliar faces (Horton et al., 2008; Johnson et al., 2017; Johnson and Miles, unpublished), the spatial position of dots (Turcotte et al., 2005; Couture and Tremblay, 2006; Tremblay and Saint-Aubin, 2009; Guérard et al., 2011), the spatial position of sounds (Parmentier et al., 2008; Lafond et al., 2010), odors (Johnson et al., 2013), and touches (Johnson et al., 2016). Furthermore, recent work from our laboratory (Johnson et al., 2017) demonstrates that the visual Hebb repetition effect exhibits a number of characteristics consistent with those identified by Page et al. (2013) for verbal memory. Specifically, that learning is both greater when the filler and Hebb trials comprise different items (see also Melton, 1967; Smalle et al., 2016; cf. St-Louis et al., 2018), and evident when the interval between repetitions of the Hebb sequence is increased. Taken together, these findings point persuasively to the Hebb repetition effect as a generalized feature of order memory.

The similarity of the Hebb repetition effect across a range of stimulus types contrasts starkly with the observation that inverted face stimuli fail to elicit the Hebb repetition effect (Horton et al., 2008). In their study, Horton et al. reported the Hebb repetition effect for upright faces under conditions of both quiet and concurrent articulation (a finding replicated by Johnson and Miles, unpublished). For inverted faces however, the effect was abolished, despite both upright and inverted faces producing the canonical bowed serial position function characteristic of serial order reconstruction (SOR) (e.g., Avons, 1998; Parmentier and Jones, 2000; Smyth et al., 2005; Ward et al., 2005; Guérard and Tremblay, 2008). These findings led Horton et al. (2008) to argue that the psychological distinctiveness of the stimuli may impact the rate of learning for the stimuli.

This proposed role of psychological distinctiveness was premised on earlier work (Hay et al., 2007) demonstrating qualitatively different serial position functions for yes/no recognition of upright unfamiliar-faces, inverted unfamiliar-faces, and abstract matrices. Specifically, upright faces produced extended recency, whereas both inverted faces and matrices produced reduced single item recency. Hay et al. (2007) argue that such disparities in the serial position function are due to differences in the psychological distinctiveness of the stimuli; a concept that relates to the “psychological distance between items” (p. 177), specifically, the perceived similarity between items. Given that faces are frequently encountered, it is suggested that we are highly proficient in discriminating between faces, and utilize a specialized multidimensional space for their recognition (Hay et al., 2007). Consequently, due to this highly sensitive discriminatory system, each face is represented in a sparsely populated region of psychological space, resulting in reduced interference between the face stimuli. In contrast, due to our limited experience and familiarity with inverted faces and abstract matrices, a non-specialized and relatively insensitive system is employed, which is less proficient in differentiating between these stimuli, i.e., leading to low levels of psychological distinctiveness between items. Indeed, it is worth noting that Horton et al. (2008) reported impaired SOR for the inverted faces relative to upright faces, consistent with the contention that inverted faces are perceived as more visually similar and, therefore, are more confusable. However, unlike Hay et al. (2007), qualitative differences in the serial position function for upright and inverted faces were not evident, a finding that Horton et al. (2008) attributed to task demand differences. Nevertheless, Horton et al. (2008) argue that inverted faces do not produce a Hebb repetition effect due to our inexperience with this particular stimulus type, and this results in an impaired ability to encode the items distinctively.

However, the purported role of psychological distinctiveness in determining the Hebb repetition effect is equivocal. For example, the Hebb repetition effect has been found for olfactory stimuli (Johnson et al., 2013), a stimulus type that we suggest has low psychological distinctiveness (Johnson and Miles, 2009). Moreover, it is arguable whether a dot, re-presented in different spatial locations, is a psychologically distinctive stimulus, and yet this stimulus type reliably demonstrates the Hebb repetition effect (Turcotte et al., 2005; Couture and Tremblay, 2006; Tremblay and Saint-Aubin, 2009; Guérard et al., 2011). Indeed, more generally, the absence of the Hebb repetition effect for inverted faces (Horton et al., 2008) appears anomalous given the ubiquity of the effect across other stimulus types.

Nevertheless, notwithstanding the type of stimuli used, it is evident that visual distinctiveness is an important variable in determining the visual Hebb repetition effect because increased similarity between the filler and Hebb sequences reduces the effect (Johnson et al., 2017; Johnson and Miles, unpublished). Specifically, in these studies, the visual Hebb repetition effect was evident when the filler and Hebb sequences comprised different faces (hitherto referred to as no-stimulus-overlap) but abolished when they comprised the same items (hitherto referred to as full-stimulus-overlap). This is broadly consistent with the findings for verbal stimuli where the Hebb repetition effect is moderated by full-stimulus-overlap (Page et al., 2013; Smalle et al., 2016; cf. St-Louis et al., 2018).

## Experiment 1

The present study examines the role of distinctiveness in determining the visual Hebb repetition effect. We revisit the extent to which learning of the Hebb sequence is evident when the sequence comprises stimuli of low psychological distinctiveness (as defined in Hay et al., 2007). Specifically, in Experiment 1, we explore whether inverted faces demonstrate a Hebb repetition effect. We follow closely the procedure described by Horton et al. (2008), with four important changes. First, Horton et al. (2008) reported an absence of the Hebb repetition effect for inverted faces with a relatively small sample size (*n* = 18). Whilst it is worth noting that this was sufficient statistical power to detect the effect with upright faces (under conditions of both quiet and CA), if the Hebb repetition effect is smaller for inverted faces, an increased sample size may be able to detect the effect. Therefore, in the present experiment, a sample size of 40 is used.

Second, in Horton et al. (2008), there were 6 repetitions of the Hebb sequence for each stimulus type. The Hebb repetition effect is typically assessed across 10 repetitions; it is therefore possible that there was insufficient exposure to the inverted face Hebb sequence for learning of the sequence to be detected. To address this issue, we employ 10 repetitions of the Hebb sequence spread equally across 30 trials.

Third, we manipulate between-sequence distinctiveness, by comparing the effects of full-stimulus-overlap and no-stimulus-overlap with inverted faces. Our working hypothesis is that the Hebb repetition effect is a universal feature of memory (given the range of stimuli with which this effect has been demonstrated). If this effect is common across stimuli, one might predict that features of this effect might also be generalized. To the extent that between-sequence distinctiveness promotes the Hebb repetition effect (due to minimal interference between the filler and Hebb sequences), we predict a Hebb repetition effect for no-stimulus-overlap only (consistent with that found for upright faces, Johnson et al., 2017; Johnson and Miles, unpublished; and that found with verbal stimuli, Page et al., 2013; Smalle et al., 2016).

Fourth, as highlighted during the review process, it is possible that absolute serial position recall is too rigid a scoring criterion to fully capture evidence of sequential learning. For example, if the sequence “A B C D E” was recalled as “E A B C D,” using absolute positional recall scoring, the trial would receive a score of zero. In contrast, the Levenshtein scoring procedure [Levenshtein, 1966; as used by Kalm and Norris (2016), to investigate the verbal Hebb repetition effect], examines the string similarity between the presented sequence and the recalled sequence, assessing the number of changes (i.e., the edit distance) needed to transform the recalled sequence into the original to-be-remembered sequence. Specifically, the aforementioned recalled sequence of “E A B C D,” only requires a single edit (by switching ‘E’ to the last serial position) to reproduce the original sequence (i.e., edit distance = 1). Consequently, in contrast to absolute positional scoring, this outputted sequence would demonstrate high similarity with the to-be-remembered sequence. It is therefore possible that the failure to detect learning of the inverted face Hebb sequence in Horton et al. (2008) was a result of learning being masked by the rigid scoring protocol. In the present study we examine evidence for the Hebb repetition effect using the more traditional measure of absolute serial position but also include the Levenshtein edit-distance scoring protocol as a less constrained measure of sequence learning.

### Methods

#### Participants

Forty Bournemouth University Psychology undergraduates (mean age = 21.38 years; 32 female and 8 male), participated in exchange for research participation credits. Ethical approval was obtained from the Bournemouth University Psychology Ethics Committee.

#### Materials

The to-be-remembered sequences were presented on a 23 inch (58.4 cm) Hewlett-Packard (Palo Alto, CA, United States) Elite Display E231 monitor using the experimental software E-prime 2.0 (Psychology Software Tools, Inc.).

The unfamiliar-face stimuli were selected at random for each participant from a corpus of 60 faces (taken from Facial Recognition Technology, FERET, database (Phillips et al., 1998). Each face comprised 52 mm × 64 mm frontal images of Caucasian males lacking both facial hair and eye-wear. Images were grayscale and elliptically cropped to remove hair and ears.

For the full-stimulus-overlap condition, 5 faces were selected at random (and rotated 180°). These were used in the construction of both the filler and Hebb sequences. For the no-stimulus-overlap condition, 15 faces were selected at random (and rotated 180°). Five faces were selected to construct the Hebb sequence, 5 to construct one filler sequence, and 5 to construct the other filler sequence.

#### Design

A 4-factor (2 × 2 × 10 × 5) within-participants design was employed with the factors stimulus-overlap (no-stimulus-overlap and full-stimulus-overlap), sequence type (filler and Hebb), experimental epoch (1–10), and serial position (1–5). Each experimental epoch comprised 3 trials; 1 Hebb and 2 filler trials. The experiment was blocked by stimulus overlap condition, with the order of blocks counterbalanced across participants.

#### Procedure

Participants were tested individually in a quiet laboratory booth and seated facing the computer at a distance of 60 cm. Each participant completed two blocks of 30 trials, preceded by three practice trials. Each trial was initiated by a keyboard press and comprised the sequential presentation of 5 inverted faces, each displayed for 1000 ms with a 1000 ms inter-stimulus-interval (ISI). Following a 1000 ms retention interval (RI) the test phase commenced. At test, the 5-inverted faces from the preceding sequence were re-presented simultaneously on the screen in a circular array. The position of each inverted face in the test array was randomized across both trials and participants. To recall, the participant was required, using the mouse, to reconstruct the presentation order of the preceding sequence by clicking on each stimulus in order. Once selected, the stimulus acquired a blue border signifying stimulus selection, and the participant was unable to either change or repeat a selection.

The test-phase was self-paced and successive trials did not commence until the five stimuli from the previous sequence had been selected. The experiment lasted approximately 30-min.

### Results

**FIGURE 1:**
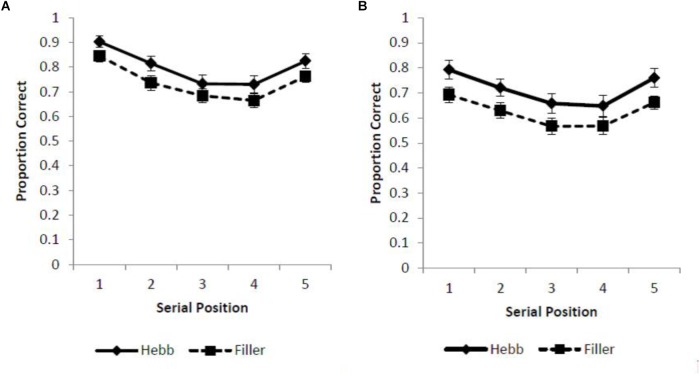
**(A,B)** Mean proportion correct recall scores for the filler and Hebb sequences of inverted faces as a function of serial position (1–5) under conditions of **(A)** full-stimulus-overlap and **(B)** no-stimulus-overlap. Error bars represent the standard error of the means.

Two different scoring criteria were applied to sequence recall (with analysis computed using JASP, JASP Team, 2018). First, a strict scoring criterion was adopted such that a response was recorded as correct only if the item was recalled in the serial position of its original presentation (absolute positional recall). Using this scoring protocol we report standard serial position functions for the filler and Hebb trials across the overlap and no overlap conditions (Figures 1A,B).

The second scoring criterion was based upon the Levenshtein edit distance procedure (Levenshtein, 1966; Kalm and Norris, 2016). For each sequence we examined how many edits were required to transform the recalled order into the original to-be-remembered order. For example, if the five inverted faces were recalled as 5th, 1st, 2nd, 3rd, and 4th, the edit distance = 1 (i.e., a single edit of moving the 5th item to the end of the recalled sequence reproduces the original sequence). However, if the five inverted faces were recalled as 5th, 4th, 3rd, 2nd, and 1st, four edits are needed to transform the outputted order into the correct sequence. Each recalled sequence therefore had an edit distance that varied between 0 and 4. As described by Kalm and Norris (2016), the Levenshtein recall score is then derived by dividing the edit distance by the sequence length (in this instance 5) and subtracting that number from 1.

**FIGURE 2:**
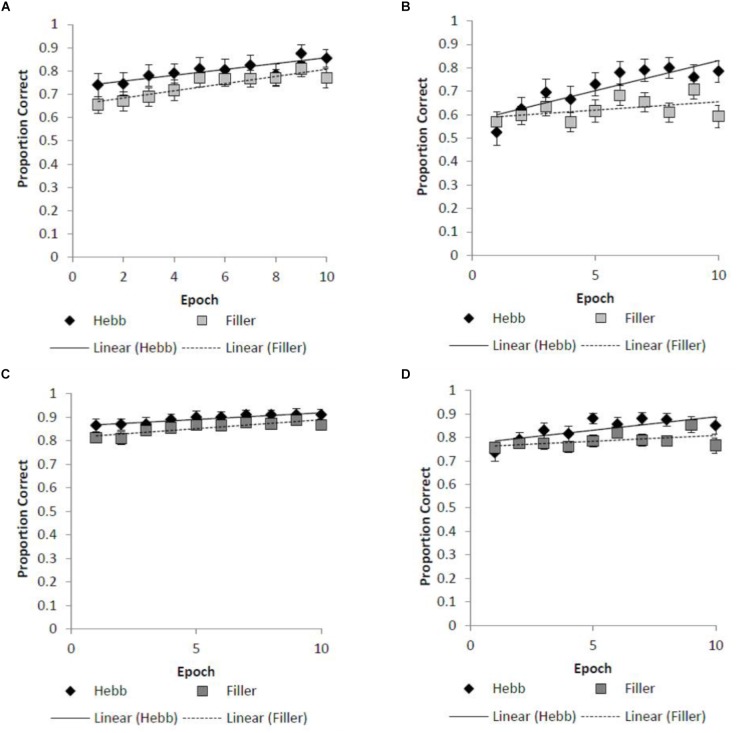
**(A–D)** Mean improvement for the filler and Hebb inverted face sequences as a function of experimental epoch (1–10) for the **(A)** absolute position recall scoring under conditions of full-stimulus-overlap, **(B)** absolute position recall scoring under conditions of no-stimulus-overlap, **(C)** Levenshtein edit distance scoring under conditions of full-stimulus-overlap, **(D)** Levenshtein edit distance scoring under conditions of no-stimulus-overlap. Line of best fit depicts the learning gradient for both sequence types. Error bars represent the standard error of the means.

For both the absolute positional recall and Levenshtein scoring protocols, the Hebb repetition effect was assessed by fitting the recall data to a linear regression model for each participant in order to compute individual learning gradients for both the filler and Hebb trials. We then contrast learning gradients for the filler and Hebb trials across the different stimulus overlap conditions. Figures 2A–D shows the learning gradients for the filler and Hebb trials for each stimulus type as a function of experimental epochs across the overlap and no overlap conditions. This is shown for the absolute positional recall (Figures 2A,B) and Levenshtein (Figures 2C,D) scoring protocols.

#### Absolute Positional Recall

A 2-factor (2 × 2) within-participants ANOVA was computed with the factors stimulus overlap (full-stimulus-overlap and no-stimulus-overlap) and sequence type (filler and Hebb). The main effects of both stimulus overlap [*F*(1,39) = 0.202, *p* = 0.656, $\eta_{p}^{2}$ = 0.005] and sequence type [*F*(1,39) = 1.925, *p* = 0.173, $\eta_{p}^{2}$ = 0.047] were non-significant. Importantly, the stimulus overlap by sequence type interaction was significant [*F*(1,39) = 6.111, *p* = 0.018, $\eta_{p}^{2}$ = 0.135]. Further analysis (including Bayes Factors computed using default priors, JASP Team, 2018) revealed that the interaction is explained by superior learning for the Hebb sequence in the no-stimulus-overlap condition only [*t*(39) = 3.050, *p* = 0.004, *d* = 0.482, BF_10_ = 8.811; mean gradient for filler and Hebb conditions = 0.009 and 0.026, respectively]. For the full-stimulus-overlap condition, there was no evidence of the Hebb repetition effect [*t*(39) = -0.709, *p* = 0.482, *d* = -0.112, BF_10_ = 0.216; mean gradient for filler and Hebb conditions = 0.013 and 0.018, respectively]. That is, we show strong evidence in support of the Hebb repetition effect under no-stimulus-overlap and strong evidence in favor of the null following full-stimulus-overlap.

#### Levenshtein Edit Distance

The same 2-factor (2 × 2) within-participants ANOVA described above was performed on the Levenshtein edit distance scoring of sequential recall. The main effects of both stimulus overlap [*F*(1,39) = 0.197, *p* = 0.659, $\eta_{p}^{2}$ = 0.005] and sequence type [*F*(1,39) = 1.044, *p* = 0.313, $\eta_{p}^{2}$ = 0.026] were non-significant. Importantly, the stimulus overlap by sequence type interaction was also non-significant [*F*(1,39) = 2.614, *p* = 0.114, $\eta_{p}^{2}$ = 0.063].

### Discussion

Experiment 1 has found evidence in support of the Hebb repetition effect with inverted faces but only under conditions of no-stimulus-overlap. That the Hebb repetition effect is abolished under full-stimulus-overlap is consistent with both upright unfamiliar-faces (Johnson et al., 2017; Johnson and Miles, unpublished) and verbal stimuli (Page et al., 2013; Smalle et al., 2016; cf. St-Louis et al., 2018), and is thought to reflect similarity-induced interference arising from the same stimuli being employed in the filler (non-repeating) sequences. Demonstration of the Hebb repetition effect with inverted faces is consistent with our working hypothesis that the Hebb repetition effect is found independent of stimulus type and directly contradicts the null effect reported by Horton et al. (2008).

It is worth noting that the Hebb repetition effect was found with absolute positional recall scoring but not using the Levenshtein edit distance protocol. It was suggested at the review stage that the Levenshtein edit distance may provide a more sensitive measure of sequential learning and therefore may provide a more subtle assessment of repetition learning with inverted faces. However, whilst visually a trend was evident in respect to sequential learning (see Figure 2D), this effect was not statistically significant. One explanation for this unexpected finding is that applying the Levenshtein scoring procedure to SOR (as was the case in the present study) is arguably less sensitive than when applied to ISR (as in Kalm and Norris, 2016). In SOR, the to-be-remembered items are re-presented at test, with participants required to select the items in the order of original presentation. Participants are forced to respond to all items, therefore only order errors are possible. For ISR, participants must recall both the items and position; therefore both item (omissions and intrusions) and order errors are possible. The reduced scope for errors in SOR results in less degrees of freedom for the edit distance. Specifically, in the present study there is a maximum number of four edits required to transform the outputted sequence into the original to-be-remembered sequence. This is contrasted with absolute positional scoring where accuracy for the sequence can range from 0 to 5. That the absolute positional scoring scale has more sensitivity may explain why the Hebb repetition effect was found only with this measure. Such a limitation should be noted for future studies applying Levenshtein edit distance to SOR.

It is not clear why our finding for inverted face sequences differs to that reported by Horton et al. (2008). The methodologies employed match very closely: both studies employed sequences of 5 faces with identical presentation times (1000 ms exposure with a 1000 ms ISI), and our serial position curves for inverted-face (see Figures 1A,B) replicated the canonical bowed SOR functions reported in Horton et al. (2008). The studies did, however, differ with respect to both stimulus cropping and number of trials. For the present study, faces were elliptically cropped (removing hair and ears). It is plausible that such stimulus cropping rendered learning of the Hebb sequence more challenging, since it was harder for participants to assign verbal labels to the external features of the faces. However, if this were the case one might have expected to report the Hebb effect to be absent in the current study and present in Horton et al. (2008), when in fact the reverse was reported.

With respect to the number of trials, the present study employed 30 trials per block (with 10 repetitions of the Hebb sequence), compared to 18 trials (with 6 Hebb repetitions) in Horton et al. (2008). It is possible therefore, that an increase in the number of Hebb trials is required in order to detect the Hebb repetition effect for inverted faces. We investigated this possibility by reanalyzing the rate of learning for inverted faces in the present experiment (under conditions of no-stimulus overlap) for epochs 1–6 only, thus equating to the number of Hebb trials in Horton et al. (2008). To the extent that the Horton et al. (2008) study required an increase in trials in order to detect the Hebb repetition effect, then we predict equivalent learning in the present study across the first 6 repetition. In contrast to this prediction, the learning gradient for inverted faces remained higher for the Hebb sequence relative to the filler sequence (=0.045 and 0.016, respectively), and this gradient of learning exceeds that reported by Horton et al. (2008) (gradient of improvement for the Hebb and filler sequences = 0.009 and -0.003, respectively). We therefore conclude that number of trials employed cannot account for the different effects found with inverted faces.

## Experiment 2

In Experiment 1, we have shown strong evidence in support of the Hebb repetition effect being shown using inverted faces (under conditions of no-stimulus-overlap). This contradicts the proposition that the effect is only found with ‘psychological distinctive’ stimuli (Horton et al., 2008). In Experiment 2, we test the role of psychological distinctiveness more directly by comparing the Hebb repetition effect for high psychologically distinctive stimuli (upright unfamiliar-faces) with that for low psychologically distinctive stimuli (inverted unfamiliar-faces and abstract matrices: as defined by Hay et al., 2007). As in Experiment 1, we also manipulate stimulus overlap to examine whether between-sequence distinctiveness moderates the Hebb repetition effect commonly across stimulus types.

To the extent that between-sequence distinctiveness promotes the Hebb repetition effect (due to minimal interference between the filler and Hebb sequences), we predict a Hebb repetition effect for no-stimulus-overlap only (consistent with Johnson et al., 2017; Johnson and Miles, unpublished). Moreover, if psychological distinctiveness affects the Hebb repetition effect (Horton et al., 2008), we further predict an interaction between stimulus type and stimulus overlap, such that under conditions of no-stimulus-overlap, the Hebb repetition effect is found but only for the high psychologically distinctive stimuli (i.e., unfamiliar faces).

### Methods

#### Participants

Ninety-six Bournemouth University Psychology undergraduates (mean age = 19.87 years; 81 female and 15 male), participated in exchange for research participation credits. Participants were randomly assigned in equal numbers (*N* = 16 per group) to one of 6 experimental conditions (full-stimulus-overlap with upright faces, full-stimulus-overlap with inverted faces, full-stimulus-overlap with matrices, no-stimulus-overlap with upright faces, no-stimulus-overlap with inverted faces, and no-stimulus-overlap with matrices). Ethical approval was obtained from the Bournemouth University Psychology Ethics Committee.

#### Materials

The face stimuli were as described for Experiment 1.

The abstract matrices were selected at random from a corpus of 60. The matrices had dimensions 32 mm by 32 mm and comprised 16 squares, of which 8 were white and 8 were black. Any matrix configurations that resembled easily nameable symbols (e.g., letters) were removed from the set.

As described for Experiment 1, for the full-stimulus-overlap condition, 5 stimuli were selected at random for each participant from one of the different stimulus types: upright faces, inverted faces (i.e., faces rotated 180°), and abstract matrices. These were used in the construction of both the filler and Hebb sequences. For the no-stimulus-overlap condition, 15 stimuli were selected at random for each participant from one of the different stimulus types: upright faces, inverted faces, and abstract matrices. Five items were selected to construct the Hebb sequence, 5 to construct one filler sequence, and 5 to construct the other filler sequence.

#### Design

A 5-factor (2 × 3 × 2 × 10 × 5) mixed design was employed with the between-participants factors stimulus overlap (no-stimulus-overlap and full-stimulus-overlap), and stimulus type (upright faces, inverted faces, and abstract matrices); and the within-participants factors sequence type (filler and Hebb), experimental epoch (1–10), and serial position (1–5). Each experimental epoch comprised 3 trials; 1 Hebb and 2 filler trials.

#### Procedure

The procedure was as described for Experiment 1, with the exception that each participant completed a single block of 30 trials only. The experiment lasted approximately 15-min.

### Results and Discussion

**FIGURE 3:**
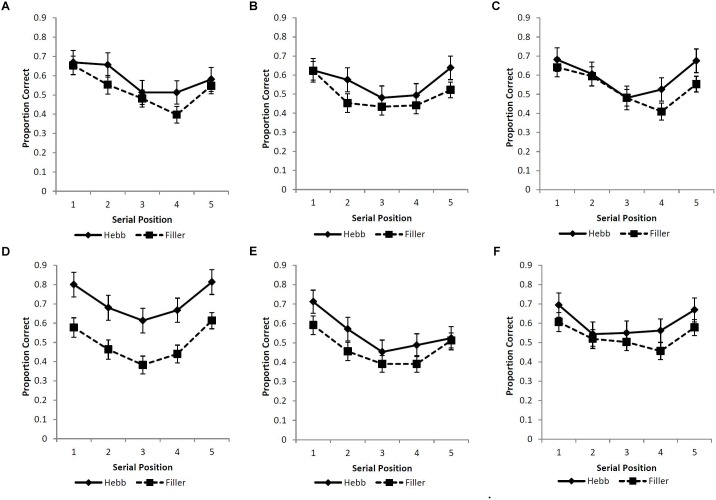
**(A–F)** Mean proportion correct recall scores for the filler and Hebb sequences as a function of serial position (1–5) for the **(A)** upright faces under conditions of full-stimulus-overlap, **(B)** inverted faces under conditions of full-stimulus-overlap, **(C)** matrices under conditions of full-stimulus-overlap, **(D)** upright faces under conditions of no-stimulus-overlap, **(E)** inverted faces under conditions of no-stimulus-overlap, and **(F)** matrices under conditions of no-stimulus-overlap. Error bars represent the standard error of the means.

A strict scoring criterion was adopted such that a response was recorded as correct only if the item was recalled in the serial position of its original presentation. Figures 3A–F displays the serial position functions for the filler and Hebb trials across the six experimental conditions. Following the findings of Experiment 1, the Levenshtein edit distance was not included.

**FIGURE 4:**
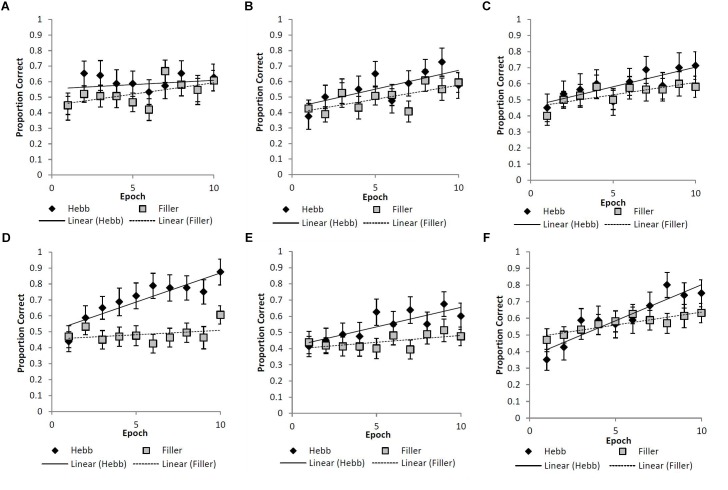
**(A–F)** Mean proportion correct recall scores for the filler and Hebb sequences as a function of experimental epoch (1–10) for the **(A)** upright faces under conditions of full-stimulus-overlap, **(B)** inverted faces under conditions of full-stimulus-overlap, **(C)** abstract matrices under conditions of full-stimulus-overlap, **(D)** upright faces under conditions of no-stimulus-overlap, **(E)** inverted faces under conditions of no-stimulus-overlap, and **(F)** abstract matrices under conditions of no-stimulus-overlap. Line of best fit depicts the learning gradient for both sequence types. Error bars represent the standard error of the means.

As described for Experiment 1, the Hebb repetition effect was assessed by fitting the recall data to a linear regression model for each participant in order to compute individual learning gradients for both the filler and Hebb trials. Figures 4A–F shows the learning gradients for the filler and Hebb trials for each stimulus type as a function of experimental epochs across the six experimental conditions. These effects are shown in more detail in Table 1 where the mean learning gradients for filler and Hebb sequences are reported across the six experimental conditions, along with the 95% CIs for the difference between filler and Hebb sequences.

**Table 1:** Mean improvement gradients for the filler and Hebb sequences across the six experimental conditions.

Stimulus overlap	Stimulus type	Sequence type		
			
		Filler gradient	Hebb gradient	Difference [95% CI]
Full	Upright faces	0.016	0.010	-0.007 [-0.027,0.013]
	Inverted faces	0.018	0.025	0.007 [-0.013,0.027]
	Matrices	0.015	0.024	0.009 [-0.011,0.029]
None	Upright faces	0.001	0.037	0.035 [0.015,0.056]
	Inverted faces	0.006	0.024	0.018 [-0.002,0.039]
	Matrices	0.015	0.043	0.028 [0.008,0.048]


*Gradient represents the mean improvement in proportion correct at each experimental epoch.*

A 3-factor (2 × 3 × 2) mixed ANOVA was computed with the between-participants factors stimulus-overlap (full-stimulus-overlap and no-stimulus-overlap), stimulus type (upright faces, inverted faces, and matrices), and the within-participants factor sequence type (filler and Hebb). The main effects of both stimulus overlap [*F*(1,90) = 0.210, *p* = 0.648, $\eta_{p}^{2}$ = 0.002] and stimulus type [*F*(2,90) = 1.028, *p* = 0.362, $\eta_{p}^{2}$ = 0.022] were non-significant. The main effect of sequence type was significant, [*F*(1,90) = 12.673, *p* < 0.001, $\eta_{p}^{2}$ = 0.123], revealing superior learning for the Hebb sequences (mean gradient for the filler and Hebb sequences = 0.013 and 0.027, respectively). Importantly, the stimulus overlap by sequence type interaction was significant [*F*(1,90) = 8.726, *p* = 0.004, $\eta_{p}^{2}$ = 0.088]. Further analysis (including Bayes Factors computed using default priors, JASP Team, 2018) revealed that the interaction is explained by superior learning for the Hebb sequence in the no-stimulus-overlap condition only [*t*(47) = 4.623, *p* < 0.001, *d* = 0.667, BF_10_ = 707.1; mean gradient for filler and Hebb conditions = 0.008 and 0.034, respectively]. For the full-stimulus-overlap condition, there was no evidence of the Hebb repetition effect [*t*(47) = 0.432, *p* = 0.668, *d* = 0.062, BF_10_ = 0.171; mean gradient for filler and Hebb conditions = 0.018 and 0.020, respectively]. This provides strong evidence for the Hebb repetition effect under no-stimulus-overlap and strong evidence for the null under full-stimulus-overlap. The two-way interactions between stimulus overlap and stimulus-type [*F*(2,90) = 1.028, *p* = 0.362, $\eta_{p}^{2}$ = 0.022], and stimulus-type and sequence type [*F*(2,90) = 0.254, *p* = 0.776, $\eta_{p}^{2}$ = 0.006] were both non-significant. It was predicted that only psychologically distinctive stimuli (i.e., upright faces) would exhibit evidence for the Hebb repetition effect and that this would be demonstrated under conditions of no-stimulus-overlap only. However, the important three-way interaction between no stimulus overlap, stimulus type, and sequence type was non-significant [*F*(2,90) = 1.172, *p* = 0.314, $\eta_{p}^{2}$ = 0.025].

In summary, Experiment 2 has shown evidence for the Hebb repetition effect across upright unfamiliar-faces, inverted faces, and abstract matrices but for the no-stimulus-overlap condition only. That we have found the Hebb repetition effect with upright faces (i.e., stimuli purported to possess high levels of psychological distinctiveness) is unsurprising and consistent with a growing number of studies (Horton et al., 2008; Johnson et al., 2017; Johnson and Miles, unpublished). However, of novelty is the reported evidence for the Hebb repetition with stimuli that are purported to possess low levels of psychological distinctiveness (inverted faces and abstract matrices, Hay et al., 2007). Our findings therefore contradict the claim that the Hebb repetition effect is moderated by the psychological distinctiveness of the stimuli.

It is, however, worth noting that the learning effects for inverted faces (under conditions of no-stimulus-overlap) were less compelling than in Experiment 1 (see Table 1 where 95% CIs for the difference marginally spans zero). Experiment 2 was less powered (with sample sizes more comparable to the original Horton et al., 2008, study) and may provide some insight into why Horton et al. (2008) failed to find the effect with inverted faces.

## General Discussion

Across two experiments we have shown evidence for the Hebb repetition effect to be found with stimuli that purportedly possess low levels of psychological distinctiveness (as defined by Hay et al., 2007). In Experiment 1, we showed strong evidence for the Hebb repetition effect with inverted faces. In Experiment 2 we report a Hebb repetition effect for upright unfamiliar faces, inverted unfamiliar faces, and abstract matrices. Importantly, across both experiments, the Hebb repetition effect was found only when there was no-stimulus-overlap across the Hebb and filler trials. That there is no Hebb repetition effect for these visual stimuli when presented in the full-stimulus-overlap condition is a finding consistent with recent work (Johnson et al., 2017; Johnson and Miles, unpublished). This lack of the Hebb repetition effect is taken to reflect the action of the filler sequence disrupting acquisition of the Hebb sequence, due to similarity-driven interference between the two sequence types. In Experiment 1, our strong evidence of a Hebb repetition effect for inverted faces directly contradicts the finding of Horton et al. (2008). Moreover, the Hebb repetition effect for abstract matrices contradicts the proposal of Horton et al. (2008) that sequences comprising stimuli of low psychological distinctiveness (as which Hay et al., 2007, categorized matrices) show impaired learning of the Hebb sequence.

Notwithstanding the difference with Horton et al. (2008) with respect to reporting a Hebb repetition effect for inverted faces, our findings are consistent with the Hebb repetition effect being observed across a range of stimulus types. Indeed, more pertinently, our findings are consistent with observations of the Hebb repetition effect using stimuli with which participants may be unfamiliar and for which, therefore, possess low psychological distinctiveness, e.g., the spatial position of dots (Turcotte et al., 2005; Couture and Tremblay, 2006; Tremblay and Saint-Aubin, 2009; Guérard et al., 2011), odors (Johnson et al., 2013), and touches (Johnson et al., 2016). This is further emphasized by our current finding of the Hebb repetition effect with abstract matrices, a stimulus type purportedly low on levels of psychological distinctiveness (Hay et al., 2007). Together, these findings suggest, therefore, that the Hebb repetition effect is not mediated by psychological distinctiveness and point to the commonality of the Hebb repetition effect across stimulus types.

In addition, the present findings replicate the recently reported effect of stimulus overlap on the visual Hebb repetition effect (Johnson et al., 2017; Johnson and Miles, unpublished). In the present study, the Hebb repetition effect was evident for upright faces, inverted faces, and abstract matrices under conditions of no-stimulus-overlap but abolished under full-stimulus-overlap. This is consistent with the proposal that the stimuli comprising the filler sequences interfere with the stimuli comprising the Hebb sequence (because both sequences comprise the same stimuli) and thus, impede the learning of the Hebb sequence. It is worth noting, however, that whilst the verbal Hebb repetition effect is weakened under full-stimulus-overlap, it is not abolished (Page et al., 2013; Smalle et al., 2016). This could be interpreted as subtle cross-modal differences in the functioning of the Hebb repetition effect. Alternatively, this disparity could be explained by differences in sequence encoding strategies driven by stimulus characteristics. Specifically, Smalle et al. (2016) argued that the verbal Hebb repetition effect survives full-stimulus-overlap because at learning the sequence is parsed into large chunks. They further contend that given it is unlikely that the sub-chunks used for remembering the filler and Hebb sequences are perfect anagrams, there is less interference (i.e., overlap) between the filler chunks and Hebb-sequence chunks. However, such a chunking strategy is arguably harder for sequences of unfamiliar-faces (compared to words) and, as a consequence, the chunk representations in memory that are purportedly formed for the Hebb sequence are perfect anagrams of those that are purportedly stored for the filler sequences. When overlapping lists are unchunked, this therefore increases the similarity between overlapping lists and hinders learning.

In summary, the present study demonstrates that (1) the visual Hebb repetition effect is evident both for sequences comprising stimuli possessing high levels of psychological distinctiveness and sequences comprising low levels of psychological distinctiveness and (2) the extent to which the stimuli comprising both the filler and Hebb sequences overlap (that is, between-sequence distinctiveness) is the key determinant in demonstrating the visual Hebb effect. In addition, our findings further contribute to the evidence showing that the Hebb repetition effect is evident cross-modally (even for stimuli for which participants have limited exposure) and that full-stimulus-overlap impedes the Hebb repetition effect across different stimulus types. Taken together, these cross-stimuli Hebb repetition effects may be interpreted as further evidence for domain general order memory effects (e.g., Hurlstone et al., 2014; Vandierendonck, 2016).

## Author’s Note

Our experimental material and anonymised data is available via the Open Science Framework at https://osf.io/whz9g/.

## Ethics Statement

The research presented in the manuscript was conducted in accordance with the ethical principles of the American Psychological Association concerning research with human participants. The protocol for the research was approved by Bournemouth University’s Science, Technology and Health Research Ethics Panel (approval reference: 5023). All participants involved in the research gave their written informed consent in accordance with the Declaration of Helsinki.

## Author Contributions

AJ contributed to the study design, collected data, performed the analysis, and drafted the manuscript. CM contributed to the study design and contributed to writing and drafting the manuscript.

## Conflict of Interest Statement

The authors declare that the research was conducted in the absence of any commercial or financial relationships that could be construed as a potential conflict of interest.
